# Nonsteroidal anti‐inflammatory drugs (NSAIDs) and prostate cancer risk: results from the EPICAP study

**DOI:** 10.1002/cam4.1186

**Published:** 2017-09-21

**Authors:** Solene Doat, Sylvie Cénée, Brigitte Trétarre, Xavier Rebillard, Pierre‐Jean Lamy, Jean‐Pierre Bringer, François Iborra, Thibaut Murez, Marie Sanchez, Florence Menegaux, Didier Ayuso, Bruno Ségui, Vincent Abd El Fattah, Alain Guillaume, Jean‐Paul Constans, Olivier Delbos, Pierre Lanfray, Damien Rizet, Etienne Cuénant, Michel Locci, Etienne Cuénant, Nicolas Drianno, Bernard Marc, Paulo Soares, Antoine Faix, Samer Abdel Hamid, Bruno Ségui, Samer Abdel Hamid, Grégoire Poinas, Laurent Cabaniols, Maxime Robert, Rodolphe Thuret, Didier Brel, Lysiane Schweizer, Philippe Nayraud, C. Lecam‐Savin, Roland Daniel, Jean Baptiste Perdigou, Chantal Compan, Mireille Granier, A Granier, Ruth Borges‐Reis, A Badsi, Jean Louis Bouzigues, Elisabeth Broquerie, Joëlle Simony, Frédéric Bibeau, Pierre Baldet, Isabelle Serre, Valérie Costes, Marie Laure Gaume, F. Montels, François, Pascal Dumas, Martine Buono, Isabelle Bonnefille, Georges Ruiz, Didier Paleirac

**Affiliations:** ^1^ CESP (Center for Research in Epidemiology and Population Health), Inserm, Team Cancer and Environment Université Paris‐Saclay, Université Paris‐Sud Villejuif France; ^2^ Hepato‐gastroenterology Department, Unit of Gastrointestinal Tumor Screening and Treatment Pitié‐Salpêtrière Hospital, Paris Public Hospital Authority (AP‐HP) Paris France; ^3^ Registre des Tumeurs de l'Hérault Montpellier France; ^4^ Service Urologie Clinique Beau Soleil Montpellier France; ^5^ Institut médical d'Analyse Génomique‐Imagenome Labosud, Montpellier France; ^6^ Polyclinique Saint Privat Béziers France; ^7^ Cabinet Urologie du Polygone Montpellier France; ^8^ Hôpital Lapeyronie Centre Hospitalo‐Universitaire Montpellier France; ^9^ Department of Epidemiology, Gillings School of Global Public Health University of North Carolina‐Chapel Hill Chapel Hill North Carolina

**Keywords:** Cyclooxygenase, inflammation, NSAIDs, prostate cancer

## Abstract

Chronic inflammation may play a role in prostate cancer carcinogenesis. In that context, our objective was to investigate the role of nonsteroidal anti‐inflammatory drugs (NSAIDs) in prostate cancer risk based on the EPICAP data. EPICAP is a population‐based case–control study carried out in 2012–2013 (*département* of Hérault, France) that enrolled 819 men aged less than 75 years old newly diagnosed for prostate cancer and 879 controls frequency matched to the cases on age. Face to face interviews gathered information on several potential risk factors including NSAIDs use. Odds ratios (ORs) and their 95% confidence intervals (CIs) were calculated using unconditional logistic regression models. All‐NSAIDs use was inversely associated with prostate cancer: OR 0.77, 95% CI 0.61–0.98, especially in men using NSAIDs that preferentially inhibit COX‐2 activity (OR 0.48, 95% CI 0.28–0.79). Nonaspirin NSAIDs users had a decreased risk of prostate cancer (OR 0.72, 95% CI 0.53–0.99), particularly among men with an aggressive prostate cancer (OR 0.49, 95% CI 0.27–0.89) and in men with a personal history of prostatitis (OR 0.21, 95% CI 0.07–0.59). Our results are in favor of a decreased risk of prostate cancer in men using NSAIDs, particularly for men using preferential anti‐COX‐2 activity. The protective effect of NSAIDs seems to be more pronounced in aggressive prostate cancer and in men with a personal history of prostatitis, but this needs further investigations to be confirmed.

## Introduction

Prostate cancer is the most common male cancer in westernized countries with an estimated 1.1 million men diagnosed with prostate cancer in 2012 worldwide 1. In France, more than 50,000 cases of prostate cancer were diagnosed in 2011, with almost 9000 related deaths that same year making it the third cause of cancer‐related mortality 2.

Except age, ethnic origin, and family history of prostate cancer that are well‐established nonmodifiable risk factors, the etiology of prostate cancer remains largely unknown. Accumulating epidemiological, biological, genetic, and experimental evidence suggested that chronic inflammation may be associated either with initiation or progression of several cancers, including prostate cancer 3, 4, 5, 6. Indeed, inflammatory cells as well as the chemokines and cytokines they produce confer a microenvironment favorable to tumor growth, by increasing the production of reactive oxygen species leading to oxidative DNA damage and reduce DNA repair, and tumor progression by promoting angiogenesis 3, 4, 5.

Regarding prostate cancer, the presence of inflammatory infiltrations localized near zones of proliferative inflammatory atrophy and prostatic intraepithelial neoplasia, considered to be potentially precancerous prostatic lesions, has contributed to reinforce the hypothesis of a possible link between chronic inflammation and prostate cancer 7. Moreover, prostate tissues with histological evidence of inflammation were more likely to develop prostate cancer than those without inflammation 8. The mechanism of inflammation is based on the action of the cyclooxygenase (COX), enzyme that catalyzes messenger molecules in inflammation pathways 9. There are two distinct isoforms of the COX enzyme: COX‐1, constitutively expressed in most tissues and involved in physiological functions, and COX‐2, expressed in several tissues during inflammation. Indeed, an overexpression of COX‐2 in human prostate cancer tissue compared to benign prostate tissue has been reported 10. Therefore, nonsteroidal anti‐inflammatory drugs (NSAIDs), used for their analgesic and antipyretic properties, and at higher doses, for their anti‐inflammatory effects, have received attention for their potential as chemopreventive drugs against cancer. They may be characterized according to their ability to preferentially inhibit COX‐1 as aspirin or COX‐2 as coxibs or oxicams 11.

Most epidemiological studies reported an inverse association between aspirin and cancer risk, especially for colorectal cancer 12. Modest inverse associations were also reported with aspirin use and prostate cancer occurrence in observational studies or meta‐analysis 13, 14, 15, 16, 17. Evidence for nonaspirin NSAIDs (NA‐NSAID) use and inverse association with cancer are even more scarce, with at least three observational studies showing even a positive association with prostate cancer risk 18, 19, 20 and others showing null or weak negative association 21, 22, 23, 24, 25, 26, 27, 28. Meta‐analysis could not give strong conclusions because of striking heterogeneity between studies 14, 15, 16, 29, with only one showing modest inverse association 16 and three of them null association.

Few of these studies provided data on type of NSAIDs and anti‐COX preferential activity, frequency, duration, recency of use, circumstances of prescription, and addressed the aggressiveness of cancer.

In this context, our main objective was to study the association between NSAIDs use (aspirin and nonaspirin NSAIDs) and prostate cancer risk based on the data from the EPICAP study.

## Materials and Methods

### Study population

EPICAP (EPIdemiology of Prostate CAncer) is a population‐based case–control study which details of the study protocol have been published elsewhere 30. Cases were men newly diagnosed with histologically confirmed prostate cancer between 2012 and 2013, aged less than 75 years old, and living in the *département* of Hérault, France at the time of diagnosis. The identification of the cases was realized by clinical research nurses, specifically trained for the study, by active search in all public and private cancer care centers of the *département*. Only cases who gave their informed consent were included in the study. Controls were men randomly selected in the general population, frequency matched to the cases, living in the *département* of Hérault as the cases, and with no history of prostate cancer at the time of inclusion. Quotas by age were established as a preliminary to yield the control group similar to the case group in terms of age in order to achieve frequency matching (5 year age group). Quotas by socioeconomic status (SES) were also set a priori to control for potential selection bias arising from differential participation rates across SES categories. These quotas by SES were calculated from the census data available in the *département* of Hérault, in order to obtain a distribution by SES among controls identical to the SES distribution among general male population, conditionally to age.

A total of 1098 cases and 1109 controls were eligible, of which 819 and 879, respectively, were included in the study representing a participation rate of 75% for cases and 79% for controls.

### Data collection

Trained clinical research nurses conducted face to face interviews of cases and controls using a standardized questionnaire based on a CAPI system (Computer‐Assisted Personal Interview). Detailed information on socioeconomic characteristics, personal medical history and drugs use, family history of cancer, diet, tobacco, and alcohol consumption, physical activity, and residential and occupational history were gathered.

Cases and controls were asked about their lifetime aspirin and NA‐NSAIDs consumption, and more specifically about the name, the frequency, and the duration of the specific NSAID they used. They were also asked about the reason of NSAID use and if the use was under medical prescription or over the counter.

For prostate cancer cases, medical information such as Gleason score or PSA at diagnosis had been extracted from their medical record or from the Hérault Cancer Registry.

### Statistical analysis

All analyses were performed using SAS 9.4 software (SAS Institute Inc., Cary, NC). NSAIDs were divided into three categories: overall users (all‐NSAIDs), aspirin users (aspirin), and nonaspirin NSAID users (NA‐NSAIDs). All‐NSAIDs users were defined as users of aspirin or NA‐NSAIDs at least once a month. Men who never took, or less than once a month, any kind of NSAIDs were considered as nonusers (reference class). The frequency of use (<1/day, =1/day, ≥1/day), duration of use (<5 years, 5–10 years, >10 years), and recency of use (former, current) were analyzed for all‐NSAIDs, aspirin, and NA‐NSAIDs users. We also classified NSAIDs according to their anti‐COX‐1 or anti‐COX‐2 preferential activity based on the known COX‐1/COX‐2 ratio of each NSAID. Coxib, diclofenac, etodolac, and oxicams inhibit preferentially COX‐2 (ratio <1), while aspirin, propionates, and indometacin inhibit preferentially COX‐1 11.

Unconditional logistic regression models were used to estimate odds ratios (ORs) and their 95% confidence intervals (CIs). Analyses were systematically adjusted for age (5‐year period), family history of prostate cancer in first‐degree relatives and race (Caucasians, others). Analyses were also adjusted for other potential confounding factors such as educational level, body mass index (BMI) (<25/25–30/≥30) or waist to hip ratio (WHR) (<0.95/≥0.95), and personal prostatitis history (no: no personal history of prostatitis/yes: at least one personal history of acute or chronic prostatitis).

Separate analyses were also conducted by prostate cancer aggressiveness according to the Gleason score (low or intermediate score ≤7 [including 3 + 4], high score ≥7 [including 4 + 3]).

### Ethics statement

Each subject enrolled in the study provides their written informed consent. The EPICAP study was approved by the Institutional Review Board of the French National Institute of Health and Medical Research (IRB‐Inserm No. 01‐040, November 2010) and by the French data Protection Authority (CNIL No. 910485, April 2011).

## Results

The characteristics of prostate cancer cases and controls are presented in Table 1. We enrolled 819 prostate cancer cases and 879 population‐based controls, and among them, age distribution in 5‐year groups was the same in both cases and controls (*P* = 0.19). Among cases of prostate cancer, 76% had a low or intermediate Gleason score ≤7 (including 3 + 4), and 22% had a high score ≥7 (including 4 + 3), that is, aggressive cancer, and among them, 10% had a Gleason score over 7. Our study population was predominantly Caucasian (>95% for both cases and controls). Family history of prostate cancer in first‐degree relatives was more frequent in cases than in controls (22% in cases vs. 9% in controls, *P* < 0.0001), consistent with the literature. Considering anthropomorphic data, BMI was similar in cases and controls (*P* = 0.79), while WHR was significantly higher in cases than in controls (73% of cases vs. 67% of controls, *P* = 0.03). Personal history of certain comorbidities, such as cardiovascular background (myocardial infarction, hypertension, stroke, diabetes) and rheumatologic background, were similarly distributed between cases and controls (*P* = 0.56, *P* = 0.52, respectively), while personal history of prostatitis was significantly more frequent in cases than in controls (*P* = 0.02).

**Table 1:** Study population characteristics

	Cases *n* = 819 (%)	Controls *n* = 879 (%)	*P*‐value^a^
Gleason score			
≤7 (3 + 4)	623 (76)	–	
≥7 (4 + 3)	180 (22)	–	
Age at diagnosis/interview, years			0.19
40–55	48 (6)	59 (7)	
55–60	99 (12)	99 (11)	
60–65	217 (27)	201 (23)	
65–70	274 (33)	285 (32)	
70–75	181 (22)	235 (27)	
Race			0.43
Caucasian	795 (97)	859 (98)	
Other	24 (3)	20 (2)	
Family history of prostate cancer at first degree			<0.0001
No	549 (67)	723 (82)	
Yes	181 (22)	77 (9)	
Educational level			0.22
No diploma	70 (9)	72 (8)	
Up to high school	376 (46)	436 (50)	
High school	113 (14)	110 (13)	
College	260 (32)	260 (30)	
Smoking status			0.077
Never	240 (29)	246 (28)	
Ex‐smoker	455 (56)	476 (54)	
Smoker	123 (15)	157 (18)	
Body mass index (BMI)			0.79
<25	231 (28)	248 (28)	
25–29	399 (49)	397 (45)	
≥30	182 (22)	207 (24)	
Waist to hip ratio (WHR)			0.03
<0.95	214 (26)	265 (30)	
≥0.95	594 (73)	590 (67)	
Prostatitis history			0.02
No	735 (90)	816 (93)	
Yes	84 (10)	63 (7)	
Cardiovascular background^b^			0.56
No	432 (53)	443 (51)	
Yes	384 (47)	427 (49)	
Rheumatologic background^c^			0.52
No	626 (78)	662 (77)	
Yes	175 (21)	202 (23)	

a Adjusted for age.

b Myocardial infarction, hypertension, stroke, diabetes.

c Rheumatoid polyarthritis, spondylarthropathies, arthrosis.

Sociodemographic and lifestyle characteristics of all‐NSAID users and nonusers among controls are shown in Table 2. NSAIDs users and nonusers were similar in terms of age, family history of prostate cancer in first‐degree relatives, educational level, and smoking status (*P* = 0.23, *P* = 0.15, *P* = 0.31, *P* = 0.35, respectively). In the contrary, NSAIDs users were more likely to have a BMI of 30 and above, a personal history of prostatitis, a cardiovascular or a rheumatologic background than nonusers (*P* = 0.002, *P* = 0.002, *P* < 0.0001, *P* < 0.0001, respectively).

**Table 2:** Characteristics of NSAIDs users and nonusers among controls

	All *n* = 879		
	Nonusers *n* = 593 (67%)	NSAID users *n* = 272 (31%)	*P*‐value^a^
Age at diagnosis/interview, years			0.10
40–55	42 (7)	13 (5)	
55–60	69 (12)	30 (11)	
60–65	141 (24)	58 (21)	
65–70	189 (32)	92 (34)	
70–75	152 (26)	79 (29)	
Race			0.06
Caucasian	583 (98)	262 (96)	
Other	10 (2)	10 (4)	
Family history of prostate cancer at first degree			0.05
No	495 (83)	215 (79)	
Yes	45 (8)	32 (12)	
Educational level			0.28
No diploma	45 (8)	24 (9)	
Up to high school	295 (50)	134 (49)	
High school	69 (11)	39 (14)	
College	184 (31)	74 (27)	
Smoking status			0.58
Never	169 (29)	76 (28)	
Ex‐smoker	320 (54)	147 (54)	
Smoker	104 (18)	49 (18)	
Body mass index (BMI)			0.002
<25	177 (30)	67 (25)	
25–29	281 (47)	106 (39)	
≥30	122 (21)	85 (31)	
Waist to hip ratio (WHR)			0.67
≤95	184 (30)	78 (29)	
>95	396 (67)	183 (67)	
Prostatitis history			0.002
No	561 (95)	241 (89)	
Yes	32 (5)	31 (11)	
Cardiovascular background^b^			<0.0001
No	333 (57)	103 (38)	
Yes	255 (43)	165 (61)	
Rheumatologic background^c^			<0.0001
No	476 (81)	175 (64)	
Yes	111 (19)	88 (32)	

a Adjusted for age.

b Myocardial infarction, hypertension, stroke, diabetes.

c Rheumatoid polyarthritis, spondylarthropathies, arthrosis.

We observed that 220 (27%) cases and 272 (31%) controls had ever used all‐NSAIDs (OR 0.77, 95% CI 0.61–0.98) (Table 3). The association decreased with the frequency of use (OR 0.93, 95% CI 0.68–1.27 for less than 1 use per day; OR 0.75, 95% CI 0.54–1.04 for 1 use per day; OR 0.38, 95% CI 0.18–0.79 for more than 1 use per day; *P* trend = 0.003) and was more pronounced for a duration of 5–10 years (OR 0.55, 95% CI 0.33–0.92), for a current use (OR 0.68, 95% CI 0.50–0.94), and when the NSAIDs use had a preferentially anti‐COX‐2 activity (OR 0.48, 95% CI 0.28–0.79).

**Table 3:** Associations between NSAIDs use and prostate cancer risk

	Cases *n* = 819 (%)	Controls *n* = 879 (%)	OR (95% CI)^a^
All‐NSAIDs			
Nonuse	596 (73)	593 (67)	1.00
Ever‐use	220 (27)	272 (31)	0.77 (0.61–0.98)
Drug issuance			
Over the counter	34 (4)	33 (4)	1.00 (0.57–1.75)
Medical prescription	181 (22)	229 (26)	0.76 (0.59–0.98)
Anti‐COX activity			
Preferential anti‐COX‐1	160 (20)	186 (22)	0.85 (0.65–1.11)
Preferential anti‐COX‐2	32 (4)	57 (7)	0.48 (0.28–0.79)
Frequency			
<1/day	109 (13)	108 (12)	0.93 (0.68–1.27)
=1/day	93 (11)	121 (14)	0.75 (0.54–1.04)
>1/day	11 (1)	30 (3)	0.38 (0.18–0.79)
Duration (years)			
<5	62 (8)	81 (9)	0.67 (0.45–0.99)
5–10	33 (4)	53 (6)	0.55 (0.33–0.92)
>10	120 (15)	133 (15)	0.94 (0.69–1.27)
Recency			
Former use	119 (15)	125 (14)	0.87 (0.64–1.18)
Current use	101 (12)	147 (17)	0.68 (0.50–0.94)
Current use ≤10 years	41 (5)	69 (8)	0.55 (0.35–0.87)
Current use >10 years	58 (7)	78 (9)	0.79 (0.52–1.20)
Former use ≤10 years	54 (7)	65 (7)	0.69 (0.45–1.06)
Former use >10 years	62 (8)	55 (6)	1.11 (0.73–1.69)

Adjusted for age, family history of cancer at first degree, race, educational level, history of prostatitis, WHR.

Associations for aspirin use and NA‐NSAIDs use are presented in Table 4. Aspirin use was slightly negatively, but not significantly, associated with prostate cancer (OR 0.86, 95% CI 0.65–1.14). Nevertheless, a frequency of 1 use per day, a duration of 5–10 years, and a current use were significantly negatively associated with prostate cancer (OR 0.59, 95% CI 0.39–0.89; OR 0.49, 95% CI 0.27–0.90; OR 0.70, 95% CI 0.48–1.00, respectively). NA‐NSAIDs use was also negatively associated with prostate cancer (OR 0.72, 95% CI 0.53–0.99), especially under medical prescription (OR 0.71, 95% CI 0.51–0.97). There was neither frequency nor duration clear effect, but current use was strongly negatively associated (OR 0.52, 95% CI 0.30–0.91).

**Table 4:** Associations between aspirin, NA‐NSAIDs use, and prostate cancer risk

	Aspirin			NA‐NSAIDs		
	Cases *n* = 819 (%)	Controls *n* = 879 (%)	OR (95% CI)^a^	Cases *n* = 819 (%)	Controls *n* = 879 (%)	OR (95% CI)^a^
Nonuse	596 (73)	593 (67)	1.00	596 (73)	593 (67)	1.00
Ever‐use	143 (17)	166 (19)	0.86 (0.65–1.14)	107 (13)	143 (16)	0.72 (0.53–0.99)
Drug issuance						
Over the counter	39 (5)	41 (5)	0.91 (0.54–1.53)	2 (0)	2 (0)	1.96 (0.17–22.7)
Medical prescription	102 (12)	120 (14)	0.86 (0.62–1.19)	100 (12)	136 (15)	0.71 (0.51–0.97)
Frequency						
<1 /day	83 (10)	58 (7)	1.39 (0.93–2.06)	49 (6)	72 (8)	0.70 (0.46–1.06)
=1/day	54 (7)	90 (10)	0.59 (0.39–0.89)	43 (5)	40 (5)	0.88 (0.53–1.45)
>1/day	4 (0)	13 (1)	0.32 (0.10–1.03)	8 (1)	18 (2)	0.53 (0.22–1.30)
Duration (years)						
<5	33 (4)	43 (5)	0.66 (0.38–1.13)	38 (5)	56 (6)	0.70 (0.43–1.13)
5–10	23 (3)	41 (5)	0.49 (0.27–0.90)	16 (2)	19 (2)	0.88 (0.40–1.93)
>10	85 (10)	81 (9)	1.14 (0.79–1.65)	49 (6)	64 (7)	0.70 (0.45–1.09)
Recency						
Former use	66 (8)	59 (7)	1.11 (0.73–1.67)	81 (10)	94 (11)	0.82 (0.58–1.18)
Current use	77 (9)	107 (12)	0.70 (0.48–1.00)	26 (3)	49 (6)	0.52 (0.30–0.91)
Current use ≤10 years	32 (4)	55 (6)	0.54 (0.32–0.90)	10 (1)	21 (2)	0.41 (0.17–1.00)
Current use >10 years	43 (5)	52 (6)	0.85 (0.52–1.40)	16 (2)	28 (3)	0.61 (0.30–1.23)
Former use ≤10 years	24 (3)	29 (3)	0.64 (0.34–1.22)	44 (5)	54 (6)	0.88 (0.55–1.41)
Former use >10 years	42 (5)	29 (3)	1.60 (0.94–2.71)	33 (4)	36 (4)	0.76 (0.44–1.31)

Adjusted for age, family history of cancer at first degree, race, educational level, history of prostatitis, WHR.

We performed all analyses according to the aggressiveness of prostate cancer (Table 5). NA‐NSAIDs use was negatively associated with aggressive prostate cancer (OR 0.49, 95% CI 0.27–0.89 vs. OR 0.78, 95% CI 0.56–1.09 for low or intermediate score); especially NA‐NSAID with preferential anti‐COX‐2 activity (OR 0.33, 95% CI 0.11–0.94). This association was not found with aspirin use.

**Table 5:** Associations between NSAIDs use and prostate cancer risk according to Gleason score

		Low‐ or intermediate‐grade prostate cancer Gleason score ≤7 (3 + 4)		High‐grade prostate cancer Gleason score ≥7 (4 + 3)	
	Controls *n* = 879 (%)	Cases *n* = 623 (%)	OR (95% CI)^a^	Cases *n* = 183 (%)	OR (95% CI)^a^
All‐NSAIDs					
Nonuser	593 (67)	446 (72)	1.00	140 (77)	1.00
Ever‐user	272 (31)	175 (28)	0.82 (0.64–1.06)	42 (23)	0.62 (0.41–0.95)
Preferential anti‐COX‐1	186 (21)	126 (20)	0.88 (0.66–1.18)	33 (18)	0.77 (0.49–1.21)
Preferential anti‐COX‐2	57 (6)	25 (4)	0.48 (0.28–0.84)	5 (3)	0.33 (0.11–0.94)
Aspirin	166 (19)	113 (18)	0.89 (0.66–1.21)	29 (16)	0.77 (0.48–1.24)
NA‐NSAIDs	143 (16)	86 (14)	0.78 (0.56–1.09)	18 (10)	0.49 (0.27–0.89)

Adjusted for age, family history of cancer at first degree, race, educational level, history of prostatitis, WHR.

Finally, stratifying on history of prostatitis (Table 6), the inverse association observed with NSAIDs was reinforced among patients with a personal history of prostatitis (OR 0.32, 95% CI 0.15–0.71) compared to men with no history of prostatitis (OR 0.85, 95% CI 0.67–1.09); especially among NA‐NSAID users (OR 0.21, 95% CI 0.07–0.59 vs. OR 0.82, 95% CI 0.59–1.12, respectively). Interaction was statistically significant for all‐NSAIDs use (*P* = 0.02).

**Table 6:** Associations between NSAIDs use and prostate cancer risk according to personal history of prostatitis

	No history of prostatitis (*n* = 1551)			History of prostatitis (*n* = 147)		
	Cases *n* = 735 (%)	Controls *n* = 816 (%)	OR (95% CI)^a^	Cases *n* = 84 (%)	Controls *n* = 63 (%)	OR (95% CI)^a^
All‐NSAIDs						
Nonuser	534 (73)	561 (69)	1.00	62 (74)	32 (51)	1.00
Ever‐user	198 (27)	241 (30)	0.85 (0.67–1.09)	22 (26)	31 (49)	0.32 (0.15–0.71)
Aspirin	130 (18)	152 (19)	0.91 (0.68–1.22)	13 (15)	14 (22)	0.43 (0.17–1.14)
NA‐NSAIDS	96 (13)	123 (15)	0.82 (0.59–1.12)	11 (13)	20 (32)	0.21 (0.07–0.59)

a Adjusted for age, family history of cancer at first degree, race, educational level, WHR.

All analyses were also adjusted for the reason of NSAIDs use such as cardiovascular and rheumatologic backgrounds, and results remained unchanged.

## Discussion

This study showed that NSAIDs consumption was negatively associated with prostate cancer occurrence, with a 23% reduction in prostate cancer risk, with convincing inverse associations of both aspirin and NA‐NSAIDs use. This association was more pronounced with a frequency of one or more pill a day and a current and chronic duration for all‐NSAIDs users, reaching a 52% decreased risk for anti‐COX‐2 activity users.

Our results are based on a large carefully designed population‐based case–control study conducted specifically to assess environmental and genetic factors in prostate cancer risk. Cases were identified in all cancer hospitals, either public or private, that recruited prostate cancer patients in the *département* of Hérault. In 2012, the Hérault Cancer Registry observed 770 new cases of prostate cancer, of which 575 were under 75 years of age. Considering that the number of cases observed in 2011 was identical, approximately 1150 new cases were expected during the study period (2012–2013) 31. We identified 1098 eligible cases over the study period suggesting that the recruitment of cases in the EPICAP study was exhaustive, thus limiting a potential selection bias. Even though participation rate in cases was 75%, the age distribution and the Gleason score were comparable to those of the Hérault Cancer Registry for the years 2009–2011 (private communication), which indicates that cases included in the study were representative of all eligible cases. Controls were randomly selected from the general population of the *département* of Hérault using quotas on age (5 years) to reflect the age distribution of the cases. Moreover, quotas by socioeconomic status (SES) have been established to yield the control group similar to the general population of the *département* of Hérault of the same age in terms of SES. After the selection process, we compared the distribution by SES between our control group and the male general population of the *département* of Hérault and found no significant difference, indicating that no major selection bias by SES had occurred.

To minimize misclassification of exposure, data were collected by a trained research clinical nurse using a standardized questionnaire. Many details on former and current use of NSAIDs with exhaustive questions on name of the drug, indication, prescription by a doctor or over the counter, frequency, recency, and duration of use were collected. In our study, prevalence of exposure to all‐NSAIDs was 31% among controls which was similar to other studies using questionnaires 19, 27. Recall bias is often an issue in case–control studies. Nevertheless, the use of a standardized questionnaire, a face to face interview by a research clinical nurses, and the fact that cases and controls were interviewed under the same conditions minimize the possibility of such bias. Moreover, a recall bias is usually more frequent in cases than in controls which may explain a positive association but not an inverse association as we observed in our results.

Our results were unchanged after adjustment for potential major confounding factors such as family history of prostate cancer, anthropometric indicators, or reason for NSAIDs use (cardiovascular and rheumatologic backgrounds), limiting potential confounding.

Detection bias, meaning that patient under NSAIDs would have had more frequent contact with doctors and therefore more cancers detected could not be excluded, but it could have only weakened the association as it would have gone toward positive association. In the same way, reverse causation bias would have been very unlikely to occur as it would have also gone toward a positive association.

Finally, an indication bias may have occurred if the reason for NSAIDs use (cardiovascular and rheumatologic backgrounds) was associated to prostate cancer. To our knowledge, there is no evidence of such an association in the literature.

Many observational studies investigated the potential protective effect of NSAIDs in cancer and there is striking evidence in colorectal cancer through the diminution of adenomas incidence (preneoplastic state) in group of patients taking NSAIDs, especially anti‐COX‐2 or aspirin 32, 33, 34. In prostate cancer, results were less convincing especially among nonaspirin NSAIDs users. Considering aspirin use, our results were consistent with those of recent meta‐analyses that reported a modest 5–15% reduction in prostate cancer risk associated with aspirin use 12, 14, 15, 16, 17. A recent cohort study observed that low dose of aspirin was associated with a decreased risk of prostate cancer (HR 0.64, 95% CI 0.48–0.86) especially after at least 5 years of use (HR 0.42, 95% CI 0.21–0.91) 35. The Cancer Prevention Study II Nutrition Cohort also showed that daily aspirin use for at least 5 years was associated to a 15% risk reduction of prostate cancer 36.

Regarding NA‐NSAIDs, results are less convincing in the literature. Our results are consistent with three studies reaching statistical significance with inverse association (OR 0.71, 95% CI 0.58–0.86) 21, 23, 24 with neither duration nor frequency‐related trends. A study using the Danish registry nationwide data found a slightly increased prostate cancer risk among NA‐NSAIDs users (OR 1.13, 95% CI 1.10–1.15) 35, concurring with two Finnish studies, one nationwide 37 and one conducted within the Finnish Prostate Cancer Screening Trial 20, that found an increased risk of prostate cancer associated with NA‐NSAIDs use, but no apparent trends with either increasing cumulative dose or duration of NA‐NSAIDs use, not supporting causal relationship between NA‐NSAIDs use and prostate cancer risk. All these positive associations could have been due to reverse causation bias and detection bias, exposing to the risk of false‐positive associations. Also, administrative data often lack information about confounding factors and sometimes no latency is taken into account. Due to important heterogeneity in studies, meta‐analysis had failed to show inverse associations of NA‐NSAIDs use on prostate cancer 14, 15, 16, 29.

Considering grade of cancer, we reported a decreased risk of aggressive prostate cancer occurrence in patients using all type of NSAIDs (high Gleason score, OR 0.62, 95% CI 0.41–0.95 vs. intermediate or low Gleason score, OR 0.82, 95% CI 0.64–1.06). This is in agreement with one study finding significant inverse association of daily aspirin taken in the 12 previous months with highly aggressive tumors with Gleason score ≥7 or Stage III or IV (OR 0.88, 95% CI 0.78–0.99 vs. less aggressive tumors OR 0.94, 95% CI 0.85–1.04) and another showing significant inverse association with all type of NSAID use and tumors with Gleason score ≥7 (OR 0.80, 95% CI 0.64–0.99) versus no association for Gleason score <7 (OR 0.90, 95% CI 0.77–1.04). The Danish study showed no real trend considering Gleason score 35. Regarding anti‐COX‐2 activity, no special effect was found in the literature 38, except one case–control study focusing on coxibs that found a 55% significant decreased risk of prostate cancer, which is consistent with our findings 39.

Interestingly, the inverse association observed with all‐NSAIDs use was more pronounced in men with a personal history of prostatitis reinforcing the hypothesis that inflammation provides favorable conditions for carcinogenesis 40, 41.

In conclusion, our study provides convincing evidence that a frequent and chronic NSAIDs use decreased the risk of prostate cancer, especially aggressive prostate cancer. The specific decreased risk observed among men with a personal history is an additional evidence that targeting chronic inflammation may help preventing prostate carcinogenesis.

## Conflicts of Interest

All authors have completed and submitted the ICMJ form for disclosure of potential conflicts of interest. No financial disclosures were reported.
